# Measurement under uncertainty: theory-measurement relations in early electrophysiological research

**DOI:** 10.1007/s40656-025-00708-z

**Published:** 2025-12-01

**Authors:** Maria Şerban

**Affiliations:** Department of Philosophy, School of Politics, Philosophy, and Area Studies, University of East Anglia, Norwich Research Park, AHB 0.21, Norwich, NR4 7TJ UK

**Keywords:** Measurement, Mechanism, Electrophysiology, Experiment, Methodological pluralism, Theory-dependence

## Abstract

Recent work in philosophy of measurement has converged on a "theory-dependence consensus”, according to which measurement reliability requires sophisticated theoretical scaffolding. This consensus has been largely shaped by case studies from physics and high-precision metrology. This paper questions whether this consensus adequately captures measurement practices in biology, where researchers often operate under significant uncertainty about their target phenomena. Through detailed historical analysis of early electrophysiological research—from Carlo Matteucci through Emil du Bois-Reymond, Hermann von Helmholtz, and Ludimar Hermann—I examine how quantitative measurement practices emerged under theoretical uncertainty. The cases reveal recurring patterns including productive theoretical inadequacy and instrumental constraint-driven discovery, supporting an analytical framework that distinguishes multiple levels of theoretical involvement in measurement. Building on these cases, I argue that biological measurement practices function productively as strategies for causal discovery, and theoretically inadequate frameworks prove epistemically valuable by structuring empirical inquiry to reveal previously unrecognised causal factors.

## Introduction

Discovering how neurons communicate through rapid electrical signals was key to understanding nervous system function, from simple reflexes to complex cognitive processes.^1^ Our modern characterisation of nerve impulses as action potentials—as self-propagating waves of electrical activity along nerve fibres—emerged from over two centuries of systematic developments in experimental and theoretical practices across several scientific domains, including biochemistry, physics, and physiology (Clarke & Jacyna, 1987; McComas, 2011). Yet the path from the earliest detection of bioelectrical phenomena to quantitative measurement of neural activity was neither linear nor theoretically straightforward.

Investigating electrical phenomena in living systems presented distinctive methodological challenges whose analysis can inform contemporary debates about the relationship between measurement and theory in the life sciences. In the late 18th and early nineteenth centuries, scientists like Luigi Galvani, Carlo Matteucci, and Emil du Bois-Reymond sought to detect, measure, and describe the electrical activity associated with nerve and muscle function. Their efforts faced multiple obstacles: how to reliably detect very small electrical signals from biological tissues, how to distinguish these signals from experimental artefacts, and how to relate the measured quantities to underlying physiological processes and mechanisms.

The methodological challenges faced by these pioneering electrophysiologists raise broader epistemological questions that extend beyond this historical context. How do scientists establish reliable measurement procedures when studying poorly understood phenomena? What role does theoretical knowledge play in developing and validating quantification efforts? How does instrument development and refinement influence theoretical interpretations of measurement data?

While these epistemological questions have long concerned philosophers and historians of science (e.g., Chang, 2004; Galison, 1987; Hacking, 1983; Holmes, 1985; Latour & Woolgar, 1986; Lenoir, 1986), contemporary philosophy of measurement has increasingly converged on the view that theoretical knowledge plays a central—indeed, indispensable—role in reliable measurement practices (Mitchell et al., 2017; Parker, 2017; Tal, 2013; van Fraassen, 2008). This emerging consensus suggests that reliable measurement requires substantial theoretical scaffolding to guide instrumentation, data interpretation, and experimental design (Ohnesorge, 2023). However, this picture—largely drawn from examples in physics and high-precision metrology—may not adequately capture the distinctive epistemic dynamics of measurement in biology, where researchers often operate under conditions of uncertainty about the phenomena they study and limited experimental control over the relevant causal factors.

This paper questions whether the prevailing view that theoretical sophistication is necessary for measurement reliability proves adequate for understanding the epistemology of biological measurement. Through a detailed analysis of early electrophysiological research—tracing the trajectory from Carlo Matteucci's detection of demarcation currents through the instrumental innovations and quantitative measurements of a group of influential German physiologists (Emil du Bois-Reymond, Hermann von Helmholtz and Ludimar Hermann)—I argue for a more nuanced understanding of the theory-measurement relationship in biology.

My analysis thus addresses two interrelated issues. First, I examine whether the theory-dependence consensus adequately captures measurement practices that develop under theoretical uncertainty—a challenge that, while not unique to biology, has been downplayed in the philosophical literature focused on high-precision metrology. Second, I argue that biological measurement presents a revealing case for this analysis because it combines theoretical uncertainty with distinctive features of living systems: their historicity, variability, context-dependence, multi-scale causal complexity, and limited experimental controllability (cf. Montévil, 2019). The historical development of electrophysiology thus serves as a strategic test case where both conditions converge.

My central claim is that while measurement instruments can be theory-dependent in non-trivial ways, this dependence does not prevent the data they generate from supporting or challenging specific theoretical hypotheses. Rather than requiring a “declaration of independence” of measurement from all theory (Kosso, 1989), what is needed is a fine-grained analytical framework that distinguishes between different levels or types of theoretical involvement in biological measurement practices. I propose that in contexts where researchers possess limited knowledge about complex biological systems and cannot control many relevant causal factors—even under laboratory conditions—measurement must rely on preliminary theoretical assumptions that deliberately abstract from and idealise features of the target phenomenon. I also aim to show that these assumptions, though often crude and eventually shown to be inadequate in important ways, nevertheless play a key role in justifying the fruitfulness of quantitative measurement efforts.

More specifically, I argue that the epistemic value of theoretical assumptions in these situations lies not in their accuracy but in their capacity to enable tests that reveal which previously unrecognised factors significantly influence the phenomenon under investigation. This perspective suggests that measurement and discovery in biology proceed hand in hand—quantitative measurement programs can serve as causal discovery strategies.

This analysis has broader implications for understanding scientific progress in the life sciences. Early electrophysiologists navigated tensions between fragmentary theoretical knowledge and quantitative measurement, revealing a productive methodological pluralism that involved using multiple measurement techniques and provisional theoretical frameworks to investigate complex biological phenomena. This differs crucially from epistemic pluralism which implies the retention of multiple competing explanatory frameworks (cf. Bolinska, 2022). Instead, the electrophysiological cases show how methodological diversity enabled theoretical convergence. Multiple investigative approaches and revisable assumptions facilitated the transition from electromagnetic to electrochemical explanations rather than requiring their permanent coexistence. This perspective acknowledges the distinctive challenges of measuring living systems while remaining compatible with theoretical unification ambitions pursued in biology.

The paper proceeds as follows. Section 2 reviews current debates in philosophy of measurement, establishing the emerging consensus about the centrality of theory in measurement practices and examining how this consensus has been shaped by particular case studies from the physical sciences. Section 3 presents the historical case study of early electrophysiological measurement, demonstrating the complex dialogue between instrument building, theory development, and quantitative measurement in this field. Section 4 articulates an analytical framework that provides a nuanced account of theory-dependence in biological measurement, distinguishing different levels of theoretical involvement and their respective epistemic functions. Section 5 reflects on the broader implications of this diachronic evaluation of measurement in biology, examining how methodological pluralism can enable fruitful causal discovery under conditions of theoretical uncertainty. I conclude in Sect. 6 by considering how this analysis might inform contemporary debates about measurement practices in the life sciences.

## A theory-dependence consensus in philosophy of measurement

Measurement outcomes are typically granted distinctive epistemic authority in scientific practice, serving as reliable evidence for evaluating theories and guiding scientific reasoning. Recent philosophical work argues that this epistemic privilege is fundamentally explained by measurement's deep theoretical embeddedness rather than its theory neutrality (Mitchell et al., 2017; Parker, 2017; Tal, 2012).^2^ This emerging consensus represents a radical shift from earlier views that linked measurement's special epistemic status to its supposed independence from theoretical assumptions (e.g., Bridgman, 1927; Campbell, 1928).

This “theoretical turn” in the philosophy of measurement has been driven by several influential accounts that emphasise the indispensable role of background theories and models in measurement practices. For instance, van Fraassen (2008, pp. 179–180) has characterised measurement as a process that “allows [agents] to gather information” where “the outcome of a measurement provides a representation of the entity (object, event, process) measured, selectively, by displaying values of some physical parameters that—*according to the theory governing this context*—characterise that object” (emphasis added). For van Fraassen, theoretical and modelling assumptions are explicitly required for measurement to function as “an empirical information-gathering activity whose aim is to locate an object in a logical space.” (Ibid.)

Tal (2012) has developed this thesis further, arguing that measurement should be understood as a form of model-based inference where “a necessary precondition for the possibility of measuring is the specification of an abstract and idealised model of the measurement process.” (Tal, 2012, p. 17) In Tal's account, measurement processes are theory-dependent at multiple levels. Theoretical models play a key role in (i) guiding the interpretation of results, (ii) constructing inferences from instrument indications to measurement outcomes, and (iii) informing evaluations of measurement error and uncertainty.

Building on these insights, Parker (2017, p. 279) claims that measurement inference must be guided by assumptions that “cohere with background knowledge as much as possible—not just with relevant background theory but also with knowledge of interfering factors, limitations of instruments and human perception, and so on.” Finally, this view has gathered support from metrologists as well. For instance, Mari et al. (2017) have put forward a “structural interpretation” which emphasises three levels of theoretical framing necessary for reliable measurement: (i) a general theoretical model which characterises the measurand, (ii) a specific model adapting this to particular measurement contexts, and (iii) a theoretical model describing instrument interactions.

These approaches share a core commitment captured by Tal's (2013, p. 1168) claim that measurement is “considered possible only against a theoretical background with ever more accurate measurements requiring even richer theoretical backgrounds.” This theory-dependence consensus suggests that measurement's epistemic reliability derives from sophisticated theoretical scaffolding that guides instrumentation, data interpretation, and experimental design.

While much of this recent philosophical literature on measurement has been informed by detailed case studies (Chang, 2004; Sherry, 2011; Tal, 2013, 2016; Parker, 2017), the selection of examples in these debates has privileged physics and high-precision metrology. For instance, Tal’s (2016) influential work examines atomic time standards, while his model-based approach is illustrated through precision measurement of the Newtonian gravitational constant using sophisticated torsion pendulum apparatus (Tal, 2012, 2013). Van Fraassen's (2008) paradigmatic examples include temperature measurement involving complex data synthesis from multiple thermometer stations. Mari et al.'s (2017) structural interpretation account primarily examines precision mass measurement and electrical metrology standards. Other key contributors to the theory-dependence consensus focus also on highly controlled, precision measurement contexts (Morrison, 2009; Parker, 2017; Wilson & Boudinot, 2022).

What these examples share is that they typically involve sophisticated instrumentation, well-established theoretical frameworks, and extensive calibration procedures—contexts where substantial theoretical resources are readily available and measurement uncertainty can be rigorously quantified. This case study selection bias poses a challenge: Are these examples representative of all scientific measurement practices? Can measurement reliability be justified only through the complex theoretical scaffolding found in physics? Should measurement practices in other fields conform to recommendations derived from these physics-based accounts?

Recent work in philosophy of biology suggests this consensus may be gaining traction in other domains as well. For instance, Montévil (2019) has argued that reliable biological measurement requires substantial theoretical guidance, proposing that biological measurement must be “methodised by theory” to accommodate the distinctive features of living systems—their historicity, variability, and context-dependence. His account illustrates how the theory-dependence consensus could be extended from physics to other scientific domains.

Beyond theoretical uncertainty, biological measurement faces distinctive challenges that compound interpretive difficulties. Biological phenomena occur in systems—living organisms—that exhibit inherent variability across individuals, developmental stages, and experimental preparations. Unlike measurements in physics where samples can be arbitrarily controlled or standardised, biological researchers must contend with irreducible organismal differences. The electrophysiology cases reveal how choices about species, preparation methods, and experimental conditions significantly influenced measurement reliability and interpretability in ways absent from physical measurement. This biological variability interacts with theoretical uncertainty in ways that distinguish biological from purely physical measurement contexts.

These distinctive features of biological systems are undeniable. However, before we conclude that they *therefore* require the kind of theory-dependent account Montévil advocates—or that the theory-dependence consensus adequately captures biological measurement—we need to examine its assumptions more carefully. It is worth questioning whether the concentration of examples from contemporary metrology and established physical measurement practices may actually encourage an overly restrictive view of measurement's epistemic functions (cf. Ohnesorge, 2023). If measurement's epistemic privilege depends fundamentally on rich theoretical backgrounds, how should we understand measurement practices that develop under conditions of theoretical uncertainty or disagreement? How do we account for cases where measurement seems to proceed successfully despite limited theoretical understanding of the systems being studied?

These questions are particularly pressing for biological measurement. Even Montévil's theory-dependent account acknowledges that biological phenomena possess distinctive features that make them challenging targets for high-precision measurement. This points to a broader characteristic of research in the life sciences: investigators often operate under conditions of significant uncertainty about biological systems and limited experimental control over relevant causal factors.

This characteristic of biological research suggests that the historical development of measurement practices in biology—such as in early electrophysiological research—offers an opportunity to examine whether the theory-dependence consensus adequately captures measurement's epistemic role across different scientific contexts. By analysing how quantitative methods developed under theoretical uncertainty, we can assess whether alternative approaches might provide better insights into the relationship between measurement and theoretical understanding in biology.

## Early electrophysiological measurements: historical case studies

To examine whether the theory-dependence consensus reflects the epistemology of biological measurement practices, I turn in this section to analysing the historical development of electrophysiological measurement from the 1830s through the 1870s. This period offers a suitable testing ground for the philosophical thesis because it encompasses the transition from qualitative observations of bioelectrical phenomena to systematic quantitative measurement, while researchers operated under conditions of significant theoretical uncertainty about the underlying mechanisms. The case studies trace how physiologists in Italy and Germany developed measurement techniques, formulated and tested hypotheses, and navigated conflicts between empirical findings and theoretical expectations. By examining these historical episodes in detail, I assess how measurement and theoretical understanding interacted during a formative period in biological science. This includes analysing the instrumental choices, experimental designs, and interpretive strategies employed by the scientists involved.

### Carlo Matteucci investigates bioelectrical phenomena

The path from Luigi Galvani's initial observations of “animal electricity” to systematic quantitative measurements of electrical activity in muscles and nerves spanned several decades and involved significant methodological innovations. Galvani's foundational experiments (1791) demonstrated that dissected frog legs contracted when touched by metallic instruments, leading him to propose that living tissues possessed an intrinsic electrical “fluid” analogous to that found in electric fish. When Alessandro Volta challenged these findings by attributing the effects to contact between dissimilar metals, Galvani responded by experimentally demonstrating muscle contractions using purely biological circuits—with nerve tissue itself serving as the conductor (Galvani, 1937; Moruzzi, 1996; Piccolino & Bresadola, 2013). Despite this vindication of “animal electricity” as a genuine biological phenomenon, further progress in understanding bioelectrical mechanisms required the development of more sensitive detection methods and systematic quantitative approaches that were not yet available to Galvani.

Leopoldo Nobili's work (1828–1830) marked an important early transition toward more quantitative approaches to ‘animal electricity’ phenomena. Nobili replaced Galvani's ‘galvanoscopic leg’—a sensitive nerve-muscle preparation that detected electrical activity through visible muscle contractions—with his newly developed astatic galvanometer, an instrument that used paired magnetic needles to achieve unprecedented sensitivity in detecting weak electrical currents.^3^ Using this instrument, he measured consistent electrical activity in frog muscle preparations, though he attributed these currents to thermoelectric effects caused by differential cooling of nerve and muscle tissues during preparation. Nobili’s interpretation aligned with the dominant physical theories of his time, particularly Thomas Seebeck’s (1821) work on thermoelectric phenomena, which had demonstrated that temperature differences between dissimilar metals could generate electrical currents. Although subsequent research would support different explanations for these effects, his work provided essential instrumental foundations for subsequent electrophysiological research.

In Italy, Carlo Matteucci would soon put these instrumental advances to use. His electrophysiological research (1836–1844) began with studies of the torpedo ray's electric organ, where he used lesion experiments to identify the specific brain region controlling electric discharge—the “electric lobe” or fourth lobe of the brain stem (Matteucci, 1836, 1837). He demonstrated that this discharge could be reflexively activated through spinal pathways, establishing neural control mechanisms for bioelectrical phenomena. This work with specialised electric organs led Matteucci to investigate whether similar electrical properties might exist in ordinary muscle tissue—a question that would require the sensitive measurement capabilities provided by galvanometric techniques.

To test whether electrical activity existed in other animal muscle tissues, Matteucci initially employed Galvani's galvanoscopic frog preparation—which, working as a highly sensitive nerve-muscle detector, revealed electrical activity through visible muscle contractions. These qualitative experiments suggested that electrical phenomena were not confined to specialised electric organs, supporting Matteucci’s hypothesis that bioelectrical properties might be universal features of living tissue.

Building on this qualitative evidence, Matteucci used the astatic galvanometer to obtain quantitative measurements of electrical activity in muscle preparations. In 1842, he conclusively demonstrated the existence of “demarcation currents” flowing between injured and intact surfaces of striated muscle (Matteucci, 1842). These currents showed remarkably consistent polarity across different preparations—the injured surface was always negative relative to the intact surface—and persisted after death, indicating independence from ongoing metabolic processes. Matteucci interpreted this consistent polarity as further support for his hypothesis that all living muscle tissue possessed fundamental electrical properties analogous to those found in specialised electric organs.

**Fig. 1:**
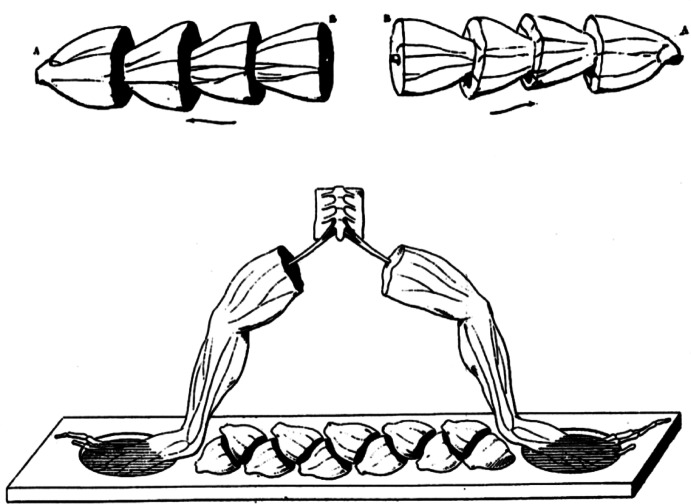
An illustration of Matteucci's experiments, showing how the halves of frog legs were connected to form sequential batteries (Matteucci, 1845a, 1845b)

While galvanometric measurements had established the existence and polarity of demarcation currents, these signals remained quite weak and sometimes difficult to detect reliably. To address this limitation, Matteucci developed his most innovative measurement technique: the “frog pile”—a biological battery constructed from sectioned frog half-thighs arranged in series (Matteucci, 1844). This method demonstrated experimentally that demarcation currents could be summed algebraically, with multiple muscle preparations generating proportionally larger electrical signals. The success of these amplification experiments appeared to provide strong empirical support for Matteucci's theoretical hypothesis. He proposed that electrical phenomena in living systems reflected the transformation of “nervous force” into electricity through specialised biological organisation, and that electric current represented the external agent with the strongest analogy to nervous activity (Matteucci, 1838, see also Moruzzi, 1996, p. 73) (Fig. 1).

Matteucci's quantitative approach also led to the discovery of an unexpected phenomenon that would prove difficult to interpret within existing theoretical frameworks. In 1842, he demonstrated that when one frog muscle contracted (through electrical or mechanical stimulation), a second frog muscle connected only through nerve contact would simultaneously contract—the “induced twitch” phenomenon (Matteucci, 1842). This experimental observation provided the first evidence of electrical signals accompanying muscle contraction that could propagate through nerve tissue to influence distant muscle preparations. However, this discovery created interpretive difficulties that reflected broader inconsistencies emerging in Matteucci's electrophysiological studies.

These challenges stemmed from contradictory observations about the relationship between muscle contraction and electrical activity. Matteucci's initial observations (1838) had suggested that muscle currents disappeared during tetanic contractions induced by strychnine, supporting the view that muscle contraction involved cessation of electrical activity. However, when he developed more sophisticated measurement techniques and repeated these experiments (1842–1844), he obtained conflicting results. Chemical stimulation of nerves sometimes produced increases rather than decreases in detectable current, while the induced twitch phenomenon suggested that muscle contraction could generate electrical signals capable of stimulating distant preparations.

To reconcile these conflicting observations while preserving his core theoretical commitments, Matteucci proposed an “action at a distance” explanation for the induced twitch phenomenon (Moruzzi, 1996). According to this hypothesis, the induced twitch resulted from some form of physical influence transmitted between muscles, rather than electrical current flow. This explanation allowed Matteucci to maintain that muscle contraction involved decreased electrical activity while accommodating the new experimental observations, but it proved difficult to test experimentally or develop into specific quantitative predictions.

The publication of Emil du Bois-Reymond's preliminary research note in 1843 introduced an alternative theoretical hypothesis that would fundamentally challenge Matteucci’s hypothesis. While du Bois-Reymond largely confirmed Matteucci's empirical findings regarding demarcation currents, he simultaneously offered a radically different explanation for the relationship between electrical activity and muscle contraction. Instead of proposing that contraction involved cessation of electrical activity, du Bois-Reymond suggested that contraction involved a temporary weakening or “negative oscillation” of the demarcation potential. This interpretation could account for the induced twitch phenomenon through direct electrical mechanisms rather than requiring mysterious “action at a distance” effects.

However, offering an alternative theoretical explanation was only the first step toward resolving the methodological challenges that had plagued electrophysiological research. Du Bois-Reymond recognised that reliable progress required addressing the fundamental problem that had undermined previous investigations: distinguishing genuine bioelectrical phenomena from experimental artefacts. His systematic approach to this challenge would establish new standards for precision and reproducibility in biological measurement.

### Emil du Bois-Reymond’s quest for precise measurements

Du Bois-Reymond's approach was grounded in an explicit commitment to “mechanistic materialism” (Finkelstein, 2003), which I will treat as fundamentally methodological rather than metaphysical. His “nearly Cartesian” view that “no forces operate in the organism other than those common to physics and chemistry” reflected his belief that reliable measurement required solving the fundamental methodological problems that had plagued earlier investigators. By treating force and matter as terms of convenience rather than ultimate realities, du Bois-Reymond developed a fruitful research strategy: if electrical phenomena in living systems were identical to those in inorganic matter, then the same measurement techniques that worked in physics should work in biology, provided that the distinctive methodological obstacles of intervening on biological systems could be overcome.

Du Bois-Reymond's first experimental goal was to reliably detect and measure the weak electrical phenomena that Galvani and Matteucci had observed, but to do so with sufficient precision to establish quantitative laws governing them. However, this seemingly straightforward task confronted what he identified as the central methodological challenge: the problem of contact electricity. Previous investigators, including Matteucci, had struggled to distinguish genuine bioelectrical phenomena from electrical artefacts generated by the contact between metal electrodes and biological tissues. As detailed in the opening volume of his *Untersuchungen über thierische Elektrizität* (1848), this problem posed a fundamental threat to the field. Without solving it, any claims about the electrical nature of nerve and muscle function remained vulnerable to a Volta-like charge that the measured currents were merely experimental artefacts rather than genuine biological phenomena.

Du Bois-Reymond's initial solution—constructing electrodes from 144 layers of Swedish filter paper soaked in saline solution—successfully enabled him to detect what he called the “fundamental phenomena” of neurophysiology: the muscle current and nerve current. Through systematic measurement across different muscle preparations, fibre orientations, and animal species, he demonstrated that both phenomena followed the same geometric relationship: current flows consistently from the exterior surface to the axial cross-section of the fibre, with the surface being positively charged relative to the cross-section. By testing this pattern in isolated nerve segments and muscle fibres across a wide range of species, du Bois-Reymond claimed that these were distinct but fundamentally related electrical phenomena governed by discoverable quantitative laws (Finkelstein, 2013; Lenoir, 1981). However, this very success revealed new experimental limitations. The filter paper electrodes required large cross-sections to achieve sufficient sensitivity, which precluded investigation of more subtle phenomena such as the polarised nerve segments that would later prove crucial for understanding the phenomenon of “electrotonus”.^4^ Thus, while each methodological advance opened new experimental possibilities, they simultaneously exposed previously unrecognised constraints.

In response to these limitations, du Bois-Reymond pursued what can be called a dual strategy of instrumental refinement. First, he sought to maximise detection sensitivity by improving upon Nobili's astatic galvanometer design, constructing what he claimed was the most sensitive galvanometer yet developed. His instrument featured a conducting wire coiled some 24,160 times about two parallel plates, with two magnetised needles—their poles facing in opposite directions to compensate for the earth's magnetic field—suspended so that one needle was above, the other within, the space separating the coil sets. This configuration could detect currents so weak that “the heat of a candle” could deflect the needle.^5^

However, this extreme sensitivity was not without its own methodological complications. The instrument's responsiveness to minute electrical changes meant that the new experimental protocols had to account for an unprecedented range of potential interference sources, from temperature variations to mechanical vibrations. More critically for understanding biological phenomena, the galvanometer's slcoorly suited for detecting rapid electrical changes—it could not register the firing of individual nerve or muscle cells or respond to rapid fluctuations in current intensity.

This temporal limitation became particularly problematic when du Bois-Reymond discovered what would prove to be his most significant phenomenon: the “negative variation” (*die negative Schwankung*)—a temporary decrease in electrical intensity (later to be understood as a decrease in the electrical potential) that accompanied muscle contraction. Here was the first direct evidence that electrical signals were involved in nerve transmission and muscle action, and yet the very instrument that enabled its detection could not reveal its temporal character.

Du Bois-Reymond solved this dilemma in an experimental intervention that used tetanic stimulation which triggered rapid repeated contractions and produced a sustained decrease in electrical signal that his galvanometer could reliably measure. This phenomenon was reproduced with remarkable consistency, du Bois Reymond claiming that it produced “needle movements of forty to seventy degrees” that he could “repeat at any time, at any place, as often as desired, without it once failing” (Du Bois Reymond, 1849, vol 2).

**Fig. 2:**
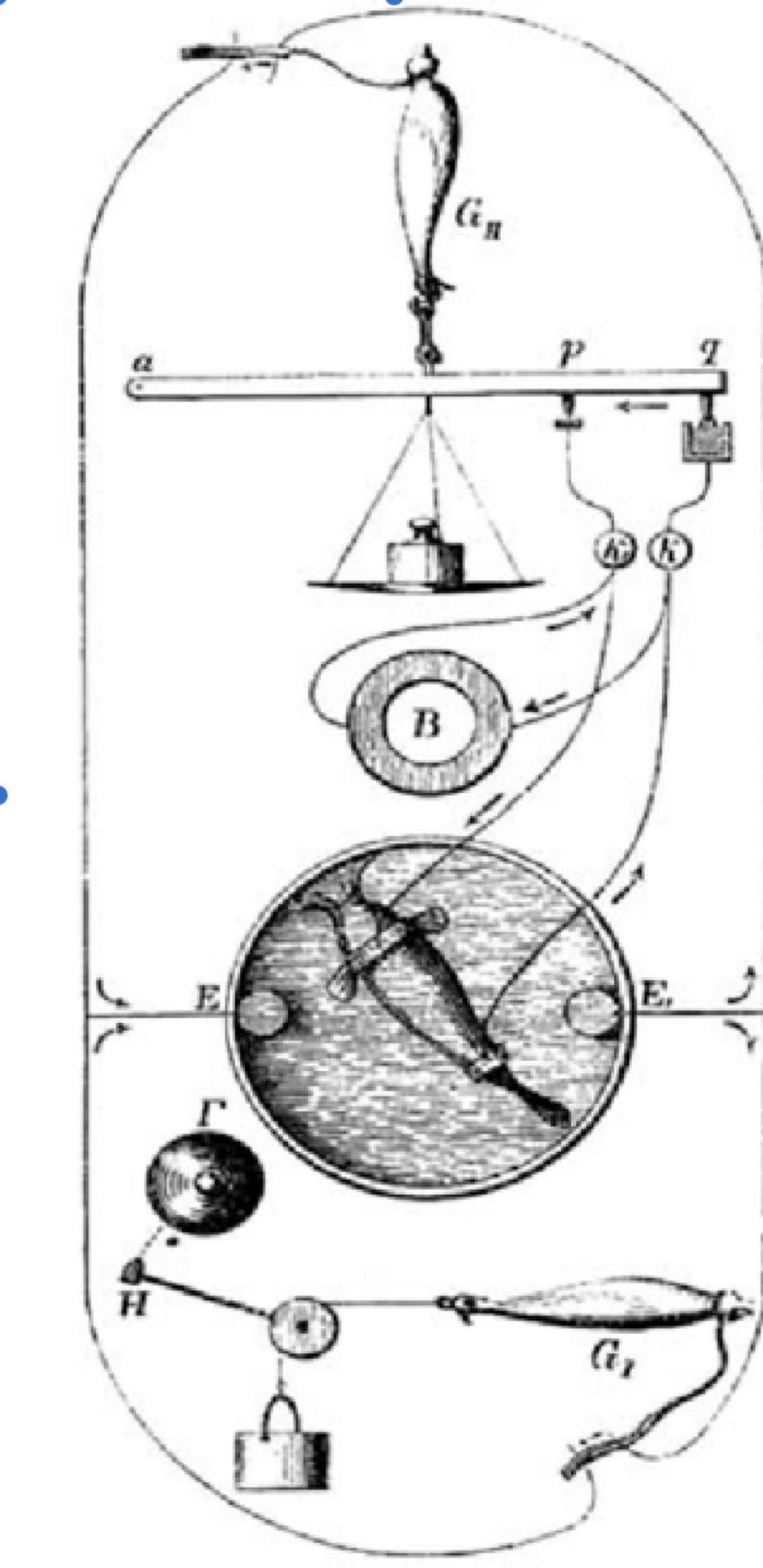
du Bois-Reymond's “physiological rheoscope” setup, showing the frog preparation (G) connected via nerve to a second preparation, with galvanometer for electrical detection. From du Bois-Reymond, *Gesammelte Abhandlungen zur allgemeinen Muskel-und Nervenphysik* (1877), Fig. 44

But demonstrating reproducible measurements was only the first step toward understanding the phenomenon's biological significance. Du Bois-Reymond conjectured that the apparently steady current registered by his galvanometer actually arose from discrete electrical events, but his instrument's temporal limitations prevented direct verification of this hypothesis. This led him to devise his most ingenious experimental solution: the “physiological rheoscope.^6^” The experimental design was elegantly simple: he connected the nerve of one muscle preparation to span between the surface and cross-section of a second muscle preparation. This arrangement meant that any electrical activity generated by the first muscle would travel through the connecting nerve to the second muscle, causing it to contract. Crucially, the second muscle's contractions provided a more sensitive detector of rapid electrical events than the sluggish galvanometer alone could offer (Fig. 2).

When the first preparation was tetanised, the second also became tetanised and remained so until stimulation ended. More revealing still, when du Bois Reymond reduced the stimulation frequency, every contraction of the first preparation produced a corresponding contraction in the second, while the galvanometer registered a continuous current reduction. He interpreted this as conclusive evidence that each muscular contraction involved a separate, individual negative variation—demonstrating the electrical nature of nerve action in a way that transcended the limitations of his primary instruments.

Taking himself to have established the electrical nature of nerve and muscle phenomena in laboratory preparations, du Bois-Reymond was ready to face the next theoretical challenge. His mechanistic framework suggested that if electrical phenomena in living systems were identical to those in inorganic matter, then the electrical regularities he had discovered in dissected preparations should also govern the functioning of intact biological systems. But this hypothesis required direct empirical verification—a technically demanding challenge given that any measurement on living systems had to contend with their baseline electrical activity.

Du Bois-Reymond's solution was ingenious: he developed a compensation method that used the subject's own body as an experimental control. By placing recording electrodes on both his right and left arms and connecting them through a galvanometer circuit, he could balance the baseline electrical activity of the two limbs against each other. When both arms were at rest, the galvanometer registered minimal deflection because the electrical activities cancelled out. However, when he voluntarily contracted the muscles of one arm while keeping the other relaxed, the galvanometer detected a clear electrical signal corresponding to the muscular activity. He took this to demonstrate that voluntary muscle contraction produced detectable electrical signals in living systems, providing the crucial bridge between laboratory findings on dissected preparations and claims about electrical activity in normal biological function (Finkelstein, 2013).

This extrapolation from laboratory to living systems measurements emboldened du Bois-Reymond to pursue a more ambitious theoretical question: if biological electrical phenomena followed the same laws as inorganic electrical activity, could they also exhibit the complex interactions that Faraday had demonstrated in electromagnetic systems? Specifically, could external electrical stimulation influence a nerve's own electrical properties, just as electromagnetic induction allowed one electrical system to influence another? To test this hypothesis, du Bois-Reymond designed experiments using an unusually long (86 mm) sciatic nerve that would allow him to apply external current to one region while measuring the nerve's own electrical activity in distant regions. When he passed a constant current through electrodes on a middle segment, he discovered that the nerve's own electrical current was systematically altered in the regions beyond the electrodes—increasing in the direction of the applied current and decreasing at the opposite end. By direct analogy to Faraday's “electrotonic state” in electromagnetic induction, he termed this condition “electrotonic,” thus establishing a first conceptual bridge between electromagnetic theory and biological electrical phenomena.

Yet these experimental successes—from the negative variation through voluntary muscle currents to electrotonus—simultaneously exposed the epistemological complexities of biological research. While du Bois-Reymond could produce consistent galvanometer deflections across all these phenomena, demonstrating that such deflections represented genuine bioelectrical activity rather than experimental artefacts remained an ongoing challenge. Alternative explanations involving perspiration, temperature changes, and residual contact effects could potentially account for the measured signals, requiring careful experimental controls that went well beyond those needed in physics laboratories (Finkelstein, 2013). The biological context introduced variables that were difficult to eliminate or control, making the interpretation of quantitative measurements inherently more complex than in inorganic systems.

So, by the late 1840s, du Bois-Reymond had established a research program that combined his commitment to mechanistic explanation with sophisticated measurement techniques capable of detecting previously unknown biological phenomena. However, this approach also revealed a fundamental tension that would characterise biological measurement more broadly: even when quantitative regularities could be established with impressive reproducibility, their theoretical interpretation required assumptions about complex biological systems that remained poorly understood. This tension between measurement capability and theoretical understanding would define subsequent developments in electrophysiology, illuminate broader questions about the relationship between quantification and explanation in the life sciences.

### Hermann von Helmholtz’s challenge

Du Bois-Reymond's mechanistic program had successfully demonstrated consistent electrical phenomena in biological systems, but it rested on a crucial untested hypothesis: that electrical phenomena in living systems were identical to those in inorganic matter and therefore should exhibit the same temporal characteristics as electromagnetic phenomena. It was Hermann von Helmholtz who recognised that this hypothesis could be tested empirically by determining whether nerve transmission occurred at speeds consistent with purely electrical explanations (cf. Olesko & Holmes, 1993).

Testing this hypothesis presented Helmholtz with a serious experimental challenge. Measuring whether nerve transmission occurred at speeds consistent with the electrical conduction hypothesis required detecting temporal intervals that were far shorter than any existing physiological methods could register. As discussed in Sect. 3.2., even du Bois-Reymond's galvanometer, with its increased sensitivity to weak currents, was too sluggish to register rapid temporal changes, while direct observation of muscle contractions could not provide the precision needed to distinguish between nearly instantaneous electrical transmission and slower biological processes.

In the winter of 1849–1850, Helmholtz addressed this challenge using an electromagnetic approach based on Claude Pouillet's (1844) galvanometer timing method.^7^ This involved stimulating a nerve at measured distances from the muscle and recording the precise time delay before contraction began through careful observation of galvanometer needle deflections. Using this approach, Helmholtz was able to establish that nerve transmission occurred at a finite velocity of approximately 27 m/s in frog nerves—a finding that posed a direct challenge to du Bois-Reymond's theoretical hypothesis. If nerve transmission were purely electrical, as the latter's explanation suggested, then signals should travel at speeds comparable to electrical conduction in metallic conductors—orders of magnitude faster than what Helmholtz actually measured.

**Fig. 3:**
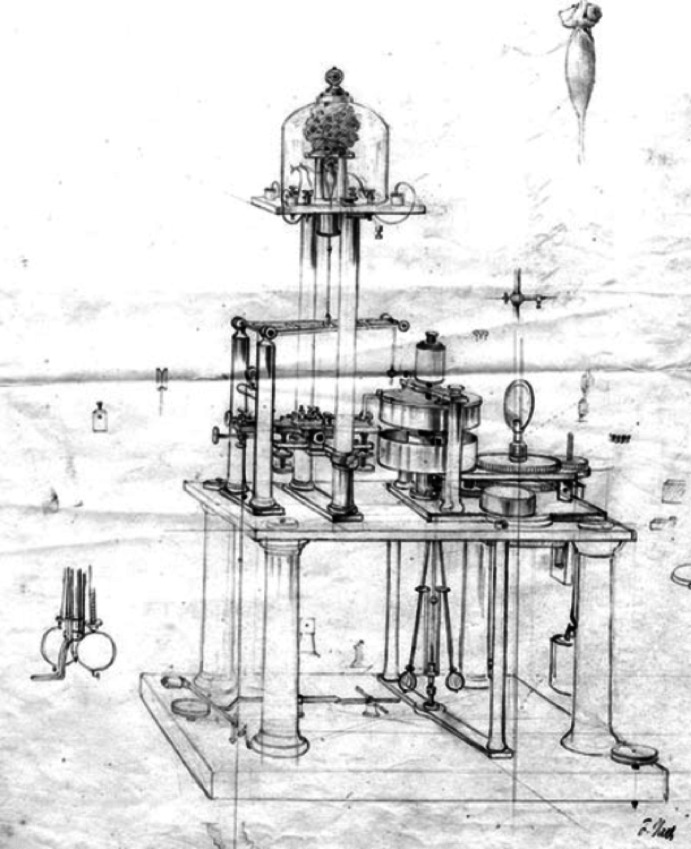
Helmholtz myograph, ca. 1860. The muscle preparation under a glass cupola is connected to a lever system with a steel needle that inscribes contractions onto a rotating glass cylinder. Drawing by Friedrich Veith, Heidelberg

Building on these temporal measurements, Helmholtz developed an influential critique of du Bois-Reymond's electro-molecular hypothesis, published in 1852. However, his initial *Preliminary Report* on these findings encountered significant communication difficulties when presented to scientific academies in Berlin and Paris (Schmidgen, 2015). These reception problems led Helmholtz to develop a new approach for demonstrating his findings. In September 1850, months after his original galvanometer measurements, Helmholtz developed his myograph—an instrument that used mechanical amplification to create visual traces of muscle contractions on a rotating drum (Fig. 3).

**Fig. 4:**
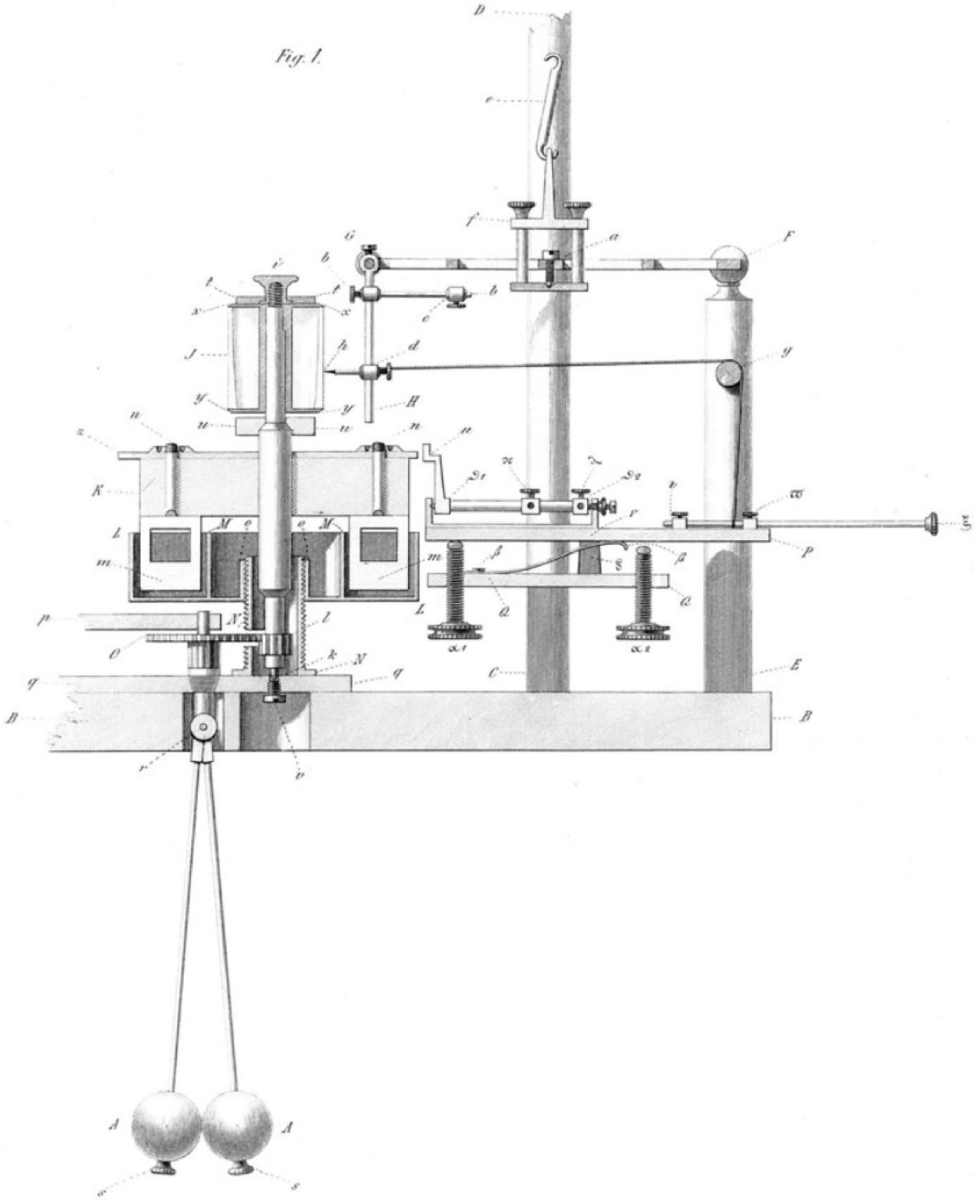
Helmholtz's myograph (1852), showing the glass cylinder, steel stylus, and rotation control mechanism. The muscle preparation and suspension apparatus are omitted from this diagram

Crucially, this instrument was designed primarily for demonstrating the principles behind measurements Helmholtz had already completed using electromagnetic methods. As Helmholtz explained to du Bois-Reymond, the myograph would allow him to “place before everyone's eyes, in 5 min, the fact of the duration of propagation in nerves in an experiment” (Kirsten et al., 1986, p. 106). The myograph's visual curves provided a compelling demonstration of the temporal delays in nerve transmission by showing the spatial displacement between traces recorded when stimulating nerves at different distances from muscles. However, as Helmholtz acknowledged, these graphic recordings were less precise than his original galvanometer measurements, noting that “the horizontal distances of the two curves cannot be measured with very great precision” (Helmholtz, 1852, p. 215) (Fig. 4).

Helmholtz's temporal measurements—obtained through galvanometer temporal methods and approximated with the myograph—revealed that nerve transmission and muscle excitation could involve electrical processes without requiring that all aspects of biological function be reducible to electromagnetic phenomena. The measured transmission speeds were more consistent with chemical or molecular processes than with the electromagnetic phenomena that du Bois-Reymond had taken as his explanatory model.

At rapid temporal scales, Helmholtz argued, electrical explanations remained compelling. His own investigations of electrotonus had revealed that nerves responded almost instantaneously to changes in applied current—rapid enough to support electrical interpretations. But this very rapidity, when contrasted with the much slower speed of nerve transmission, suggested that different mechanisms operated at different temporal scales. While electrotonus might indeed involve electrical processes analogous to those in inorganic conductors, nerve transmission appeared to require mechanisms capable of regenerating and propagating signals over long distances.

In fact Helmholtz relied on this temporal analysis to advance a broader methodological critique of du Bois-Reymond's approach. He questioned whether the electrical phenomena that du Bois-Reymond had taken as fundamental—particularly the muscle current—might themselves be artefacts of experimental manipulation. Helmholtz pointed out that it was impossible to determine whether a constant current actually existed in uninjured muscle bundles, as du Bois-Reymond's hypothesis required, since the very procedures needed to access and measure muscle tissue might themselves generate the electrical phenomena being studied.

The significance of Helmholtz's findings was immediately recognised by his contemporaries. Johannes Müller, who had previously argued that nerve transmission was too rapid and vital to be measured experimentally, found himself convinced by the results. More generally, Helmholtz’s work provided crucial evidence that biological processes involved “the rearrangement of ponderable molecules” rather than mysterious vital forces, as other members of the Berlin Physical Society had long argued (Olesko & Holmes, 1993).

Nevertheless, it would be misleading to read Helmholtz's critique as entirely destructive. As suggested above, rather than rejecting electrical explanations outright, he advocated for a more sophisticated theoretical framework that could accommodate both electrical and chemical mechanisms within a more complete account of biological function. He suggested that electrically charged particles might indeed be responsible for nerve and muscle currents, but argued that the shape and polarity of molecules—which du Bois-Reymond had elaborated in considerable detail—were “too speculative to worry about.” The key insight was that nerve transmission and muscle excitation could involve electrical processes without requiring that all aspects of biological function be reducible to electromagnetic phenomena.

This theoretical sophistication can be identified also in Helmholtz's approach to experimental design and instrumentation. Where du Bois-Reymond had focused primarily on maximising the sensitivity of current detection, Helmholtz emphasised the importance of temporal resolution and the integration of multiple measurement techniques. His work showed how thermodynamic principles could inform biological research, while his use of the myograph demonstrated how mechanical measurements could be used to investigate electrical phenomena. This methodological pluralism would prove increasingly important as electrophysiology encountered the limits of purely electrical explanations.

Helmholtz's work thus demonstrates a crucial point: quantitative measurement efforts could remain productive even when they challenge prevailing theoretical frameworks. While his temporal measurements did not directly refute du Bois-Reymond's electrical discoveries, they forced a reconceptualisation of how electrical phenomena unfolded within broader biological processes. By demonstrating that precise measurement could reveal temporal constraints that theoretical speculation had overlooked, Helmholtz can be taken to have established a new standard for biological research—one that demanded both quantitative rigour and theoretical sophistication in interpreting the relationship between measurement results and potential biological mechanisms. This approach would prove essential for navigating the increasingly complex relationship between electrical, chemical, and mechanical explanations that would characterise subsequent developments in electrophysiology.

### Ludimar Hermann and the legacy of the ‘1847 group’

The instrumental innovations and theoretical developments achieved by the 1847 group of German physiologists had successfully established electrophysiology within the camp of the quantitative sciences (Lenoir, 1986). Helmholtz's temporal measurements had introduced a new level of complexity to electrophysiological theory, suggesting that electrical and chemical processes might both be involved in biological phenomena. Yet these advances had not conclusively settled all important questions about the relationship between electrical measurements and the nature of biological phenomena. The most searching test of this relationship came from within du Bois-Reymond's own laboratory, when his student Ludimar Hermann began questioning whether the foundational phenomena of the field were genuinely biological or merely experimental artefacts (Finkelstein, 2013; Lenoir, 1986).

In 1866, Hermann began to investigate systematically whether du Bois-Reymond's key discovery—the muscle current—was indeed a property of living tissue or was an effect of the damage inflicted on muscular tissue during experimental preparation. Working with the same improved non-polarising electrodes that his teacher had developed, Hermann's growing suspicions appeared to threaten not just specific experimental findings, but the broader interpretive framework that linked electrical measurements to biological mechanisms.

Hermann's key experiment involved immersing a frog sartorius muscle partway into a saltwater bath, placing one electrode on the dry surface of the muscle and another in the saline solution. When he gradually warmed the solution to near 40 °C, the current suddenly jumped in strength. Upon cooling, the current decreased but failed to return to its original level. This was, Hermann argued, clear evidence that the electrical activity resulted from the chemical decomposition of the tissue injured by contact with the electrolyte solution.

But Hermann's critique extended far beyond questioning the muscle current. He accused du Bois-Reymond of “misconstruing his own experiments” (1878b; Hermann, 1878a) and proposed that all bioelectrical phenomena were artefacts of tissue injury during experimental preparation. According to Hermann, du Bois-Reymond had never detected any genuine biological electrical activity—only the consequences of damage inflicted by experimental procedures. If Hermann was right, then the entire electromagnetic analogy that had guided electrophysiological research required fundamental abandonment rather than mere modification.

Given du Bois-Reymond’s methodological commitments to rigorous measurement and experimentation, it should not be surprising that his immediate response was to carefully replicate Hermann's experiments. When he repeated the sartorius experiment ten times, he witnessed no initial muscle current. Warming the saline did produce a signal, but one so small that it could not be convincingly distinguished from a thermoelectrical effect—and unlike Hermann's results, the current failed to decrease when the solution cooled. However, du Bois-Reymond argued that Hermann's own interpretation suffered from several flaws (du Bois-Reymond, 1867). If surface decay was the source of electrical activity, as Hermann proposed, why did thick muscles produce stronger currents than thin ones with greater surface areas of decomposition? How could Hermann's chemical hypothesis account for the precise periodicity of tetanic contractions? And why should damaged tissue consistently behave in ways that followed predictable electrical laws rather than exhibiting the irregular patterns expected from uncontrolled chemical decay?

Drawing on his extensive investigations of muscle metabolism, Hermann countered by proposing what he called a “physiological model” that made chemical processes primary and electrical phenomena secondary (Hermann, 1878a, 1870). His detailed analysis of rigor mortis^8^ had revealed the key biochemical components—glycogen, myosin, phosphoglyceric acid, lactic acid, and carbon dioxide—and demonstrated a continual increase in myosin formation with corresponding consumption of glycogen and increased production of acid byproducts. Since analysis of active muscle revealed the same biochemical substances present during rigor mortis, Hermann concluded that both processes involved fundamentally similar chemical pathways, occurring at different temporal scales (Hermann, 1870; Lenoir, 1986).

This insight allowed him to construct what he termed the “alteration theory” of nerve transmission, which treated muscle contraction and rigor mortis as “one and the same” chemical process occurring at different temporal scales (Finkelstein, 2013). When a nerve is stimulated, he proposed, a region at the point of stimulus undergoes chemical alteration analogous to a “momentary rigor mortis.” This alteration renders the region electronegative relative to neighbouring regions, causing positive charge from adjacent sectors to flow toward the altered area and initiating similar chemical changes in neighbouring regions. This process propagates along the nerve as each altered region triggers transformations in its immediate neighbours (Hermann, 1870).

Hermann's alteration theory elegantly addressed several problems that had plagued du Bois-Reymond's electromagnetic approach. Most significantly, it could account for Helmholtz's measurements showing that nerve transmission occurred at speeds far slower than electromagnetic conduction (Lenoir, 1986). Since Hermann's model required time-consuming chemical transformations at each point along the nerve, the observed transmission speeds fit naturally within his theoretical framework rather than posing an anomaly requiring auxiliary hypotheses.

**Fig. 5:**
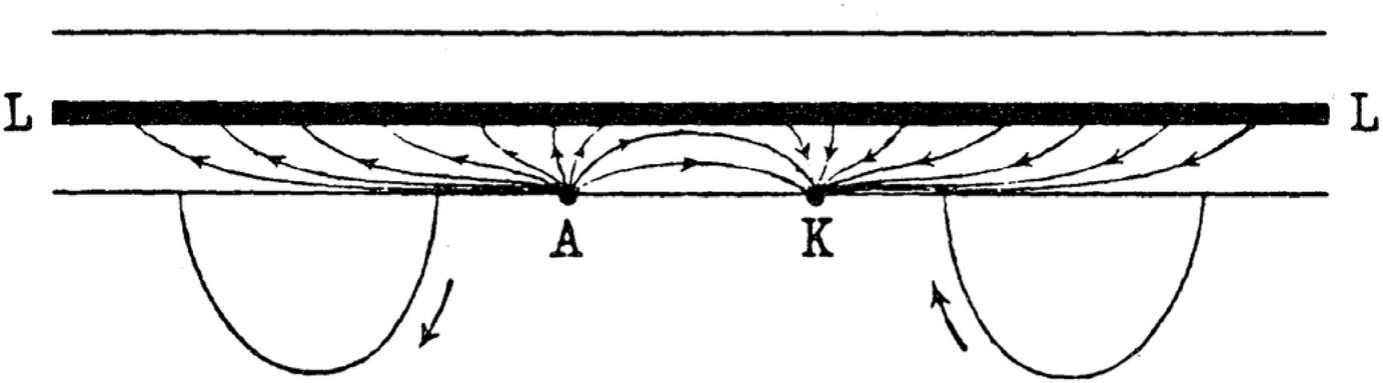
Hermann's cable model showing current flow from A to K through a conducting wire (LL) in electrolytic fluid. Current loops indicate galvanometer readings at different positions. From Hermann (1872)

To demonstrate how electrical phenomena could emerge from purely chemical processes—thus supporting his alteration theory over du Bois-Reymond's electromagnetic approach—Hermann developed his ingenious “cable model” (Hermann, 1872). This physical model consisted of a glass tube filled with dilute sulfuric acid, with a platinum wire running through the centre and capillary junctions placed at regular intervals for recording electrodes. When current was applied between two points on the tube wall, the resulting patterns of electrical activity precisely matched those observed in biological preparations under electrotonic conditions. Hermann's cable model played a key role in showing that identical electrical measurements could potentially support fundamentally different interpretations of underlying biological mechanisms (Fig. 5).

Initially, du Bois-Reymond defended his position vigorously, arguing that Hermann's chemical theory relied on “circular reasoning and ad hoc hypotheses” and failed to explain why electrotonic effects could be precisely controlled and reproduced (du Bois-Reymond, 1867). However, as Hermann's work gained support and additional evidence accumulated, du Bois-Reymond gradually modified his stance. By the end of his career, he was teaching his students that evidence seemed to favour Hermann's interpretation over his own original theory.^9^

Hermann's challenge marked a crucial turning point in the development of electrophysiology. His demonstration that electrical and chemical explanations could account for identical experimental data forced the field to develop more sophisticated approaches to the relationship between measurement and theoretical interpretation. While his specific alteration theory would eventually be superseded, his emphasis on chemical processes opened new explanatory possibilities that challenged prevailing electromagnetic frameworks. The methodological tension between electrical and chemical perspectives that he introduced proved particularly influential for subsequent electrophysiological research (Clarke & Jacynta, 1987).

## Theory-dependency in biological measurement

The historical episodes examined above reveal a complex picture of how measurement and theory interact in biological research contexts. Rather than supporting the view that measurement reliability requires sophisticated theoretical scaffolding, the trajectory from Matteucci through Hermann demonstrates epistemic dynamics of multiple levels of theoretical involvement and the productive role of theoretical inadequacy. These patterns suggest the need to revise some general assumptions underlying the theory-dependence consensus in contemporary philosophy of measurement.

### From historical patterns to analytical categories

I highlight four patterns as particularly relevant for an epistemological analysis of theory-measurement relations in biological contexts. First, the episodes analysed in Sect. 3 demonstrate what I shall call *productive theoretical inadequacy*—the capacity of theories about biological mechanisms to enable genuine discoveries that extend beyond, or even contradict, the original theoretical framework. Matteucci's “action at a distance” explanation for the induced twitch phenomenon motivated systematic investigation of nerve-muscle interactions that revealed electrical propagation effects his original framework could not accommodate. Similarly, du Bois-Reymond's electromagnetic analogy for bioelectrical phenomena generated a systematic measurement program that established the electrical nature of nerve transmission, even as subsequent research yielded evidence that could not support simple electromagnetic models.

Second, the historical cases demonstrate how the limitations of measurement instruments, rather than hindering scientific progress, drove methodological and instrumental innovations that led to the discovery of unexpected features of biological phenomena. I label this pattern *instrumental constraint-driven discovery*. Du Bois-Reymond's galvanometer could not register rapid temporal changes, yet this limitation pushed him to develop his ingenious physiological rheoscope technique, which provided the first evidence that nerve transmission involved discrete electrical events. Similarly, Helmholtz's recognition that existing physiological methods could not detect the temporal intervals needed to measure nerve transmission speeds motivated his adaptation of Pouillet's electromagnetic timing method to biological measurement. This instrumental innovation enabled him to record precise time delays through galvanometer needle deflections, achieving temporal resolution that previous physiological approaches could not provide. These measurements revealed transmission speeds that challenged purely electrical explanations.

The third pattern that emerges from the previous analysis concerns the *interpretive underdetermination* that affects biological measurement. This pattern appeared first in the analysis of Matteucci's work on the induced twitch phenomenon. When one frog muscle contracted and caused a second muscle (connected only through nerve contact) to simultaneously contract, Matteucci struggled to choose between electrical propagation and his “action at a distance” explanation, as the identical experimental observations could support either interpretation. But perhaps the most striking example is Hermann's systematic challenge to du Bois-Reymond's electrical theory. Hermann's cable model showed that electrical phenomena indistinguishable from those observed in biological preparations could arise from purely chemical processes, forcing recognition that measurement data alone could not adjudicate between competing mechanistic hypotheses. So, identical electrical measurements could support different theoretical explanations (foregrounding electromagnetic versus chemical mechanisms). This underdetermination, while prima facie problematic, proved epistemically productive by forcing researchers to develop additional experimental strategies for discriminating between competing causal hypotheses.

Finally, I believe one can find clear evidence even in these early days of electrophysiology that researchers were confronted with the *multi-scale causal complexity* of biological systems which in turn complicated their efforts to articulate the relationships between theory and measurement. Helmholtz's temporal measurements revealed that different mechanisms operated at different scales—electrical processes governing rapid phenomena like electrotonus, while slower chemical or molecular processes governed nerve transmission itself. Similarly, Hermann's alteration theory grappled with temporal complexity by proposing that muscle contraction and rigor mortis represented “one and the same” chemical process occurring at different time scales. These findings collectively indicate that biological measurement must navigate multiple mechanistic hypotheses operating at different scales as a pathway towards more unified explanatory accounts.

These four patterns—productive theoretical inadequacy, instrumental constraint-driven discovery, interpretive underdetermination, and multi-scale causal complexity—suggest that biological measurement practices operate under distinctive epistemic conditions. These conditions may not be adequately captured by the theory-dependence consensus discussed in Sect. 2. Rather than requiring coordinated theoretical scaffolding for reliable measurement, these historical cases reveal more complex dynamics where theoretical frameworks serve exploratory and enabling functions that go beyond providing accurate representations of underlying mechanisms.

It is important to qualify what is meant by “reliability” in these cases. The electrophysiology cases demonstrate that while the phenomena themselves exhibited robust qualitative patterns (polarity of demarcation currents, occurrence of negative variation, temporal delays in transmission), the quantitative precision varied considerably with preparation methods, species selection, and instrumental refinement. For instance, du Bois-Reymond's obsessive attention to experimental controls—from electrode design to temperature management—reflects recognition that biological measurement stability requires accounting for organism-specific variables that physical measurements could largely ignore. The measurements proved reliable enough to support theory-testing and discovery, even if they lacked the quantitative precision achievable in physics.

### Kinds of theory-dependency in biological measurement

These historical patterns suggest the need to reconsider the theory-dependence consensus emerging in philosophy of measurement. I share Ohnesorge’s (2021, 2023) suspicion that selection bias in the case studies chosen for philosophical analysis requires closer scrutiny. Examining additional historical episodes in the development of quantitative measurement might suggest ways to refine this consensus to better capture the epistemic dynamics of diverse measurement practices. The patterns identified in the analysis of early electrophysiological measurement suggest that we need more nuanced analytical categories that distinguish different levels and types of theoretical involvement. Such categories would provide an alternative to treating theory-dependence as a monolithic requirement for measurement reliability.

In what follows I propose a heuristic framework for analyzing the relationships between theory and measurement. Drawing on the electrophysiological cases, I identify at least three analytically distinct levels at which theoretical commitments interact with measurement practices. Though this framework's broader applicability will require testing against additional examples, it offers a useful starting point for a more nuanced analysis.

The first level concerns *enabling assumptions*—broad methodological commitments that make measurement programs possible without determining specific interpretations of measurement results. Du Bois-Reymond's mechanistic materialism functioned as such an enabling assumption—it justified applying physical measurement techniques to biological phenomena while remaining compatible with various specific explanations of bioelectrical mechanisms.

The second level involves the use of instrumental theories which comprise the assumptions built into measurement techniques themselves, including both the physical principles governing instrument function and the procedural assumptions required to distinguish genuine signals from experimental artefacts. For example, du Bois-Reymond's filter paper electrodes embodied theoretical assumptions about contact electricity, while his compensation method for measuring voluntary muscle currents relied on assumptions about baseline electrical activity. Likewise, Helmholtz's myograph embodied instrumental theories about mechanical amplification and timing relationships. These included assumptions about how lever movements could accurately represent muscle contraction onset and how temporal measurements could be converted into spatial recordings. These instrumental theories proved more durable than higher-level explanatory hypotheses, surviving theoretical transitions while continuing to enable productive measurement.vo

At the third level, I propose we can identify *interpretive frameworks*—theories about what biological mechanisms underlie observed measurement phenomena. These frameworks often exhibited the kind of interpretive underdetermination identified earlier, where identical measurement data could support competing mechanistic explanations. Matteucci's “action at a distance” explanation for the induced twitch phenomenon competed with electrical propagation explanations of the same experimental observations. Later, du Bois-Reymond's electromagnetic interpretation treated muscle currents as direct manifestations of electrical forces analogous to those in physical systems, while Hermann's chemical framework reinterpreted the same electrical measurements as secondary effects of biochemical processes. These interpretive frameworks proved vulnerable to empirical challenges and theoretical revision, with du Bois-Reymond's electromagnetic interpretation giving way to Hermann's chemical explanations, while both were eventually superseded by biochemical accounts. Yet the measurement techniques and phenomena they had identified remained stable throughout these theoretical transitions. This suggests that the revisability of interpretive frameworks does not necessarily undermine the reliability of the measurement practices they initially motivated or the empirical phenomena those practices revealed.

Thus the proposed multi-level analysis shows how theoretical inadequacy can be epistemically *productive* in biological measurement contexts. The electrophysiology cases suggest that preliminary theoretical frameworks serve crucial epistemic functions beyond that of providing accurate explanations of biological mechanisms. Instead, these frameworks enable systematic exploration of causal complexity under conditions of significant uncertainty *by* generating novel questions, motivating instrumental innovations, and revealing previously unrecognised causal factors influencing the phenomena under investigation.

Du Bois-Reymond's electromagnetic analogical hypothesis exemplifies this claim. While mechanistically incorrect, it generated a systematic measurement program that revealed regularities (the negative variation, electrotonus) that outlasted the theoretical hypothesis itself. His assumption that biological and physical electrical phenomena were identical motivated the development of sufficiently sensitive instruments to detect weak bioelectrical signals, while his mechanistic commitments guided experimental designs that could distinguish genuine biological currents from contact artefacts. Similarly, Hermann's alteration theory, though eventually superseded, productively challenged electromagnetic explanations and opened investigation of chemical mechanisms that proved essential for later biochemical approaches. Both cases illustrate how theoretical ideas or frameworks can prove epistemically valuable by generating the very evidence that leads to their revision or replacement.

More generally, this suggests a different way to approach the relationship between theoretical sophistication and measurement reliability in biological contexts. Rather than requiring accurate theoretical proposals for understanding the phenomena being measured, biological measurement may depend on theoretical frameworks because these are capable of generating questions about causal complexity. In other words, the value of such frameworks often lies not in their representational accuracy but in their capacity to structure empirical inquiry in ways that reveal previously unrecognised factors influencing the phenomena under investigation.

This multi-level analysis also clarifies an important distinction between methodological and epistemic pluralism (cf. Bolinska, 2022). The electrophysiology cases support methodological pluralism—the recognition that complex biological phenomena require multiple investigative techniques and provisional theoretical frameworks. However, they do not support epistemic pluralism—the view that multiple, competing explanatory frameworks must be permanently retained for complete understanding. As the transition from du Bois-Reymond's electromagnetic to Hermann's electrochemical explanations demonstrates, methodological diversity can enable theoretical convergence rather than requiring explanatory pluralism. This pattern indicates that biological measurement practices can serve a systematic causal discovery function rather than always testing predetermined theories.

## On the link between biological measurement and causal discovery

A diachronic analysis of measurement practices helps clarify the nature of this causal discovery function. Studying how measurement programs developed over time in electrophysiology shows that measurement served to explore causal complexity rather than being confined to testing specific theoretical hypotheses.

Matteucci's electrophysiological work illustrates this exploratory dynamic. His theoretical hypothesis about general animal electrical properties motivated systematic measurement efforts that revealed demarcation currents, leading him to develop new techniques (the frog pile) to explore these phenomena more systematically. However, his measurement approach also revealed puzzling phenomena—particularly the induced twitch—that his theoretical framework struggled to accommodate, ultimately forcing him toward the unsatisfactory ‘action at a distance’ explanation when electrical propagation theories proved inadequate. This developmental pattern demonstrates how measurement practices served both a confirmatory function (providing evidence for the demarcation current phenomenon) and a discovery function, uncovering unexpected phenomena that required further theoretical development.

This link between quantitative measurement and discovery recurred throughout the period. Du Bois-Reymond’s measurement program exemplifies how instrumental limitations drove discovery. His systematic efforts to overcome measurement constraints consistently revealed phenomena that exceeded his theoretical expectations—from the negative variation through electrotonus to discrete electrical events. This pattern demonstrates how the pursuit of measurement precision can function as a discovery strategy, revealing previously unknown aspects of biological systems rather than simply confirming theoretical predictions.^10^

Hermann’s challenge to du Bois-Reymond further demonstrates how measurement practices could serve a discovery function. Rather than simply rejecting electrical explanations, Hermann used measurement techniques to systematically explore alternative causal pathways. His sartorius muscle experiments, which showed that heating saline solutions could generate electrical signals, suggested that chemical decomposition rather than electrical properties might underlie bioelectrical phenomena. Hermann then pursued systematic biochemical measurements of muscle tissue, analysing the chemical components involved in rigor mortis and demonstrating that these same substances appeared in active muscle preparations. These quantitative biochemical investigations led him to propose his alteration theory, which treated muscle contraction and rigor mortis as related chemical processes occurring at different temporal scales. His cable model then provided a proof-of-concept, demonstrating that purely chemical processes could generate electrical phenomena indistinguishable from those observed in biological preparations, thus revealing an entirely new explanatory pathway for understanding bioelectrical phenomena.

These cases convincingly show that biological measurement practices served as systematic strategies for exploring the causal complexity of biological phenomena under conditions of theoretical uncertainty. Rather than requiring sophisticated theoretical scaffolding for reliability, these electrophysiological measurement efforts proved productive because they operated with provisional, revisable frameworks that enabled empirical exploration. This analysis suggests several important amendments to the theory-dependence consensus in contemporary philosophy of measurement. While the consensus correctly identifies the central role of theoretical assumptions in measurement practices, it may overstate the requirements for theoretical sophistication and accuracy, at least in biological contexts.

## Conclusions

Through a historical analysis of early electrophysiological research, I have traced how pioneering researchers from Carlo Matteucci through Ludimar Hermann developed systematic quantitative approaches to biological phenomena under conditions of significant theoretical uncertainty. My analysis reveals that these measurement practices operated through complex epistemic dynamics involving multiple levels of theoretical involvement—from enabling assumptions through instrumental theories to interpretive frameworks—each serving distinct epistemic functions.

The electrophysiology cases demonstrate that biological measurement can function productively as a strategy for causal discovery, with theoretically inadequate frameworks proving epistemically valuable by structuring empirical inquiry in ways that reveal previously unrecognised factors influencing biological phenomena. This discovery function reflects a methodological pluralism that enabled theoretical convergence rather than requiring permanent explanatory pluralism, as researchers used multiple provisional frameworks to explore causal complexity systematically.

These historical insights have important implications for contemporary biological measurement. The electrophysiology cases demonstrate that measurement reliability can be achieved and maintained even if accompanying theoretical frameworks turn out to be inadequate as explanations of the phenomena being measured. Du Bois-Reymond’s electromagnetic theory enabled the discovery and reliable measurement of genuine bioelectrical phenomena despite being mechanistically incorrect. This suggests that measurement reliability may depend more on the theoretical framework’s capacity to structure productive empirical inquiry than on its accuracy as a representation of underlying mechanisms.

Furthermore, the historical analysis reveals that interpretive underdetermination—rather than undermining measurement reliability—can be epistemically productive in biological contexts. Hermann's demonstration that identical electrical measurements could support competing theoretical explanations did not invalidate the measurements themselves but rather clarified the need for additional experimental strategies to discriminate between alternative causal hypotheses. This suggests that measurement practices in biology may need to accommodate persistent interpretive uncertainty rather than requiring theoretical resolution for epistemic legitimacy.

As contemporary biological sciences increasingly rely on sophisticated measurement technologies to investigate complex systems—from single-cell genomics to connectomics—recognising measurement's exploratory and discovery functions becomes crucial for understanding how quantitative approaches advance biological knowledge under conditions of theoretical uncertainty. The multi-scale causal complexity revealed by Helmholtz's temporal measurements points to a persistent challenge: if biological systems involve multiple mechanisms operating at different scales, then measurement programs may need theoretical frameworks capable of accommodating methodological pluralism rather than seeking premature theoretical unification.

For philosophy of measurement, these considerations suggest that the theory-dependence consensus, while capturing important insights about measurement practices, may reflect the particular epistemic situation of high-precision metrology and physics rather than providing generally applicable principles for scientific measurement. Biological measurement practices, developing under conditions of significant uncertainty about complex causal systems, may require different epistemic standards that emphasise exploratory capacity and causal discovery over theoretical accuracy and precision.

This points toward a methodological pluralism that recognises both the productive role of provisional theoretical frameworks and the distinctive epistemic functions of measurement in the life sciences. Such a perspective challenges philosophers of science to develop more nuanced accounts of measurement epistemology that can accommodate the distinctive challenges of investigating living systems while remaining compatible with the theoretical unification ambitions pursued in biology.

## Footnotes

1 I focus here on electrical signalling since it was the primary conceptual framework available to early electrophysiologists and remains central to understanding neural function today. Modern neuroelectrophysiology recognises that neural communication involves both electrical propagation within neurons and chemical transmission between them, but chemical synaptic transmission was not established until Otto Loewi's experiments in the 1920s (Loewi, 1921, 1924) and remained controversial until mid-century (Dale, 1934; Eccles, 1964; McComas, 2011; Bechtel, 2022).

2 For instance, picking up on this surprising consensus, Ohnesorge (2023, p. 1396) notes that “the role of theory in measurement has been one, if not *the*, dominant theme in recent metrological and philosophical work on the subject.”

3 Nobili's astatic galvanometer employed two magnetised needles oriented in opposite directions and suspended together, which cancelled out the effect of Earth's magnetic field while doubling the instrument's sensitivity to weak electrical currents. This design would later be adopted and refined by Carlo Matteucci and later electrophysiologists (cf. Moruzzi, 1996).

4 Du Bois-Reymond discovered this phenomenon by passing a constant current through electrodes placed on a middle segment of a long (86 mm) sciatic nerve, observing that the nerve's own electrical current increased at one end and decreased at the other, in regions beyond where the external electrodes were placed. He coined the term by analogy to Faraday's “electrotonic state”—the idea that current-carrying conductors put surrounding matter into a “peculiar condition” that influences electrical behaviour. The phenomenon gained greater precision through Eduard Pflüger's subsequent work with non-polarising electrodes (1859), which revealed that the nerve becomes polarised between the stimulating electrodes themselves, with the region near the anode becoming positive and that near the cathode becoming negative. Electrotonus later became central to understanding nerve excitability and the mechanisms by which electrical stimulation triggers nerve impulses, representing a key bridge between electromagnetic theory and biological phenomena (cf. Lenoir, 1981).

5 Du Bois-Reymond, *Untersuchungen über thierische Elektrizität*, vol. 1 (Berlin, 1848), 160–250. The technical details of the galvanometer construction, including the 24,160-coil configuration and the claim about sensitivity to ‘the heat of a candle,’ are all documented in this section.

6 Du Bois-Reymond's term. A rheoscope in 19th-century electrical terminology was an instrument used to detect the presence and direction of electrical currents. By calling his experimental arrangement a “physiological rheoscope,” du Bois-Reymond was drawing an explicit analogy between his use of a biological preparation (the second nerve-muscle setup) as a current detector and conventional electrical detection instruments. The term reflects his innovative adaptation of electrical terminology to describe a biological experimental method that could detect rapid electrical events with greater sensitivity than his galvanometer alone.

7 Schmidgen (2015) shows how Helmholtz drew on mathematical precision techniques developed by Gauss and Weber for torsion balance measurements, allowing him to transform galvanometer needle deflections into precise time measurements rather than relying on Pouillet's empirical tables.

8 Rigor mortis refers to the post-mortem stiffening of muscle tissue caused by biochemical changes after death. Hermann studied this phenomenon systematically by analysing the chemical composition of muscle tissue at various stages during the onset of rigor mortis, comparing the concentrations of key metabolic components over time (Hermann, 1878b).

9 The resolution of this debate eventually came through Julius Bernstein's membrane theory, which provided a synthesis incorporating elements of both Hermann’s and Du Bois-Reymond’s approaches. Bernstein rescued the latter’s belief in “the preexistence of electrical differences in muscles and nerves,” but reinterpreted this in terms of electrical potential rather than current flow, and grounded it in the ion chemistry that Hermann had championed. Additional studies of temperature dependence convinced Bernstein that Hermann's metabolic hypotheses were partially correct, while parts of Hermann's explanation of the negative variation figured into Bernstein's final account of nerve signals (de Palma & Pareti, 2011; Finkelstein, 2013).

10 This pattern of measurement-driven discovery under theoretical uncertainty extends beyond nineteenth-century electrophysiology. Güttinger and Love (2025) identify parallel dynamics in contemporary functional genomics, where measurement strategies similarly serve exploratory and discovery functions in contexts facing practical or theoretical limitations, supporting the broader applicability of the framework developed here. See Güttinger and Love (2025) ‘The epistemic strength of proxies in scientific practice’, *European Journal for Philosophy of Science* 15(3):41; and their earlier analysis of modENCODE methodology (PhilSci Archive preprint 18367).
